# Underwater noise levels in UK waters

**DOI:** 10.1038/srep36942

**Published:** 2016-11-10

**Authors:** Nathan D. Merchant, Kate L. Brookes, Rebecca C. Faulkner, Anthony W. J. Bicknell, Brendan J. Godley, Matthew J. Witt

**Affiliations:** 1 Centre for Environment, Fisheries & Aquaculture Science (Cefas), Lowestoft, UK; 2 Marine Scotland Science, Aberdeen, UK; 3 Environment and Sustainability Institute, University of Exeter, Penryn, UK; 4 Centre for Ecology and Conservation, University of Exeter, Penryn, UK

## Abstract

Underwater noise from human activities appears to be rising, with ramifications for acoustically sensitive marine organisms and the functioning of marine ecosystems. Policymakers are beginning to address the risk of ecological impact, but are constrained by a lack of data on current and historic noise levels. Here, we present the first nationally coordinated effort to quantify underwater noise levels, in support of UK policy objectives under the EU Marine Strategy Framework Directive (MSFD). Field measurements were made during 2013–2014 at twelve sites around the UK. Median noise levels ranged from 81.5–95.5 dB re 1 μPa for one-third octave bands from 63–500 Hz. Noise exposure varied considerably, with little anthropogenic influence at the Celtic Sea site, to several North Sea sites with persistent vessel noise. Comparison of acoustic metrics found that the RMS level (conventionally used to represent the mean) was highly skewed by outliers, exceeding the 97^th^ percentile at some frequencies. We conclude that environmental indicators of anthropogenic noise should instead use percentiles, to ensure statistical robustness. Power analysis indicated that at least three decades of continuous monitoring would be required to detect trends of similar magnitude to historic rises in noise levels observed in the Northeast Pacific.

Underwater noise pollution can have adverse effects on marine organisms^1^,^2^,^3^,^4^ and appears to be correlated with global economic growth^5^. Sources of noise such as shipping^6^, pile driving^7^, geophysical seismic surveys^8^,^9^, and naval sonar operations^10^ have each been linked to detrimental effects on marine fauna. Long-term measurements from deep-water sites in the Northeast Pacific have shown that low-frequency (<~50 Hz) noise levels in that ocean basin increased markedly (by ~10 dB) between the 1960 s and mid-1990 s^11^,^12^. Since then, this trend has largely levelled off or begun to decline^13^, and data from the past decade suggest that more recent trends in noise levels for other ocean basins may also be mixed^14^. The extent to which these open-ocean trends apply to the shallower continental shelf seas where human activity is concentrated is unclear^15^, since long-term datasets for these regions are lacking.

Sources of anthropogenic noise can be categorised as impulsive or continuous^16^. Each type is associated with particular effects on marine fauna, and each requires a bespoke management approach to mitigate potential impact^17^. Impulsive noise consists of brief, discrete sounds with a sudden onset, such as acoustic pulses from explosions, pile driving, or seismic airguns. Such sources can elicit acute effects on animals including permanent or temporary auditory damage^16^, physiological stress, and antipredator responses (e.g. displacement^18^). For example, short-term displacement of harbour porpoise has been reported in response to pile driving^7^ and seismic surveys^19^, and *in situ* exposure of European seabass to pile driving has been observed to induce physiological stress^20^. Such noise sources are associated with activities that are typically subject to a regulatory consenting process, and can therefore be managed through licence conditions required by these regulatory processes.

Continuous anthropogenic noise is primarily generated by shipping. Since shipping routinely crosses international boundaries, management of shipping noise requires a coordinated international response (e.g. through the International Maritime Organisation). The widespread increase in noise levels caused by continuous sources has been associated with acoustic masking of biologically important cues^18^,^21^, disruption to foraging^22^, increased physiological stress^2^,^6^, and developmental deficiencies^23^ in aquatic species. Although continuous noise sources are typically less intense than impulsive sources, the pervasive presence of continuous noise from shipping could lead to more significant and widespread effects^2^,^24^. This is of particular concern given that vessel noise can affect species that mediate important ecosystem functions^25^, as well as altering predator-prey dynamics^26^.

In response to the possible impact of anthropogenic noise on aquatic life, policymakers are beginning to develop management approaches to assess and mitigate this risk through legislative frameworks such as the European Marine Strategy Framework Directive (MSFD)^27^. However, a major constraint in managing both impulsive and continuous noise is the lack of data on current and historic noise levels. This deficit limits the ability of regulators to assess the potential impact of proposed activities through the Environmental Impact Assessment (EIA) process, and constrains target setting at larger scales. A cornerstone of the policy response to anthropogenic noise will therefore be to establish noise monitoring programmes^28^. These programmes will determine baseline noise levels against which to measure future trends, and will provide evidence to assess the efficacy of management measures. Such large-scale and long-term monitoring of underwater noise is unprecedented, and methodologies that effectively target monitoring effort and that are relevant to assessing the potential impact on marine fauna and ecosystem function have yet to be developed.

To address this need, we present the first nationally coordinated effort to characterise underwater noise levels, providing baseline measurements for UK waters. Based on the variability of noise levels observed, we discuss the targeting of monitoring effort to improve the efficiency of monitoring programmes, and evaluate the efficacy of several acoustical metrics to provide statistically robust environmental indicators of anthropogenic noise. We also assess the power of the monitoring stations to detect statistically significant trends in noise levels, which can help to ensure that policy targets are set within realistic monitoring constraints. The data we report provide a basis for tracking future trends in UK waters, and the lessons learned will be of use to future noise monitoring programmes.

## Results

### Noise sources and variability

**Figure 1:**
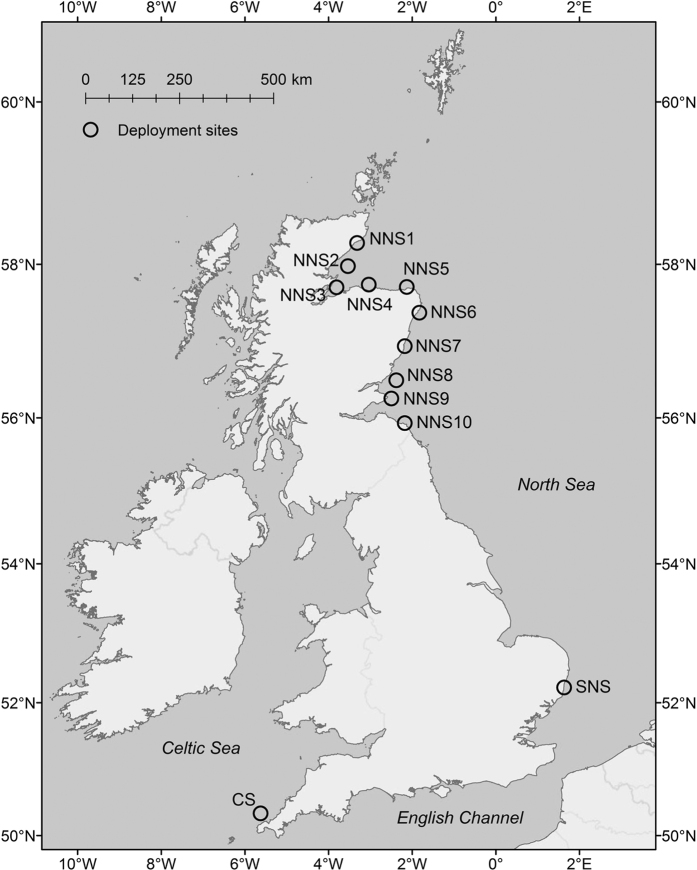
Map of monitoring locations. Abbreviations refer to monitoring area: Celtic Sea (CS), northern North Sea (NNS), and southern North Sea (SNS). Map produced using ArcGIS 10.1 (http://www.esri.com/software/arcgis/).

**Figure 2:**
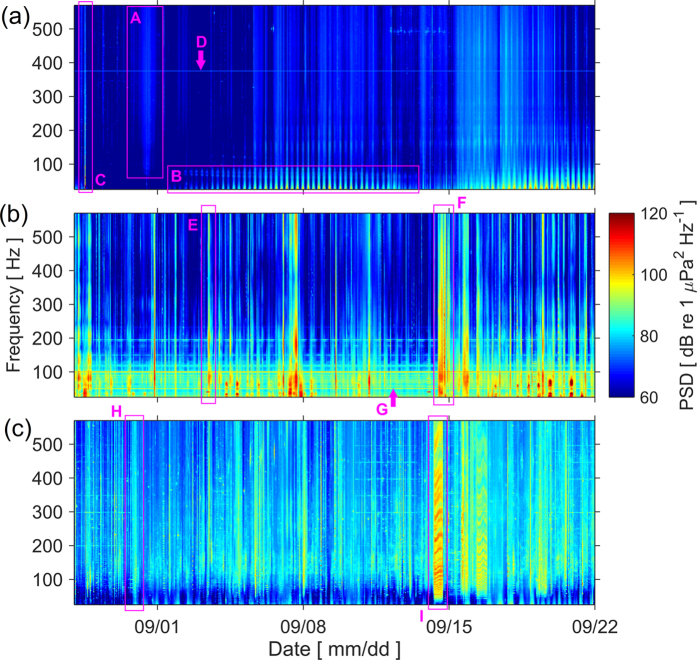
Spectrograms showing an example period during 2013. (**a**) Celtic Sea; (**b**) southern North Sea; (**c**) northern North Sea location NNS5. The colour scale indicates noise level at a given time and frequency, with absolute noise levels as indicated by the colour bar. A: example wind noise; B: tidal flow noise; C: noise from deployment vessel; D: system self-noise at 380 Hz; E: example vessel passage; F: example noise from fishing gear; G: tonal noise at 50 Hz; H: example vessel passage; I: noise from nearby moored vessel.

Variability in underwater noise levels at each monitoring location was influenced by a number of identifiable noise sources. The Celtic Sea location (Fig. 1) was the least affected by anthropogenic noise (Fig. 2a). Wind-generated noise was the primary driver of variability at this site, which appears as vertical bands of heightened noise levels above ~100 Hz (Fig. 2, label A). More intense noise levels were observed below ~100 Hz (Fig. 2, label B), and with tidal periodicity: this is ‘pseudo-noise’ caused by turbulence around the hydrophone during tidal flow^29^,^30^, and does not indicate sound present in the environment. Flow noise contamination can reduce the correlation between broadband shipping noise and the frequency bands used as anthropogenic noise indicators^31^, making these indicators less effective. Since flow noise increases with decreasing frequency^29^, lower frequency bands are more affected, particularly the 63 Hz band used for the MSFD^31^. The variability of flow noise as tidal flow rates change through the spring/neap tidal cycle can also be observed (Fig. 2a). The single distinguishable occurrence of anthropogenic noise during this period occurred on 28 August, when a vessel visited the site to redeploy the noise recorder (Fig. 2, label C), and appears as a narrow vertical band with peaks in noise level below ~100 Hz. The continuous horizontal band at ~380 Hz is self-noise from the recording device (Fig. 2, label D).

Noise levels at the southern North Sea location were influenced by human activities in the area (Fig. 2b). Fishing vessels operate at and around the monitoring location, and frequent small vessel passages were observed (Fig. 2, label E). The recordings suffered periodically from noise caused by colliding fishing gear deployed in close proximity of the recording device, which would not propagate far from the location but was prominent in the data (Fig. 2, label F). This appears as sustained periods of heightened noise levels extending throughout the frequency spectrum. The metrics used to summarise noise levels differ in their susceptibility to bias from such noise (see below), according to their robustness to outliers. Finally, tonal noise (continuous noise at a consistent frequency) from a nearby power station was observed at the 50 Hz frequency of mains electricity (Fig. 2, label G), and at various harmonics up to ~200 Hz, which highlights the possibility of onshore noise sources contributing to coastal underwater soundscapes.

A range of noise conditions were observed at the ten sites in the northern North Sea. Location NNS5 (Fig. 1) is typical of the sites with higher levels of anthropogenic noise from shipping (Fig. 2c). The spectrogram shows frequent vessel passages, which appear as intense vertical bands with peak frequencies at ~100 Hz (Fig. 2, label H), consistent with previous observations^32^,^33^. A sustained period of high noise levels on 14 September (Fig. 2, label I) appears to be a vessel moored near the monitoring location: the alternating bands of intensity are an interference pattern characteristic of the Lloyd’s mirror effect, wherein the sound travelling along a direct path to the receiver interferes with the sound reflected off the water surface. The variability in these bands corresponds to the changing water depth with tide.

### Noise level distributions

**Figure 3:**
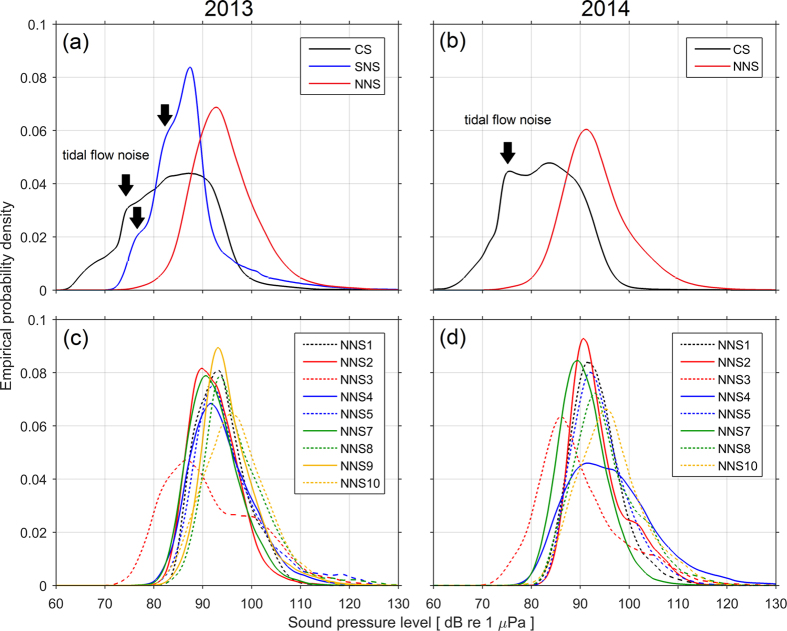
Empirical probability densities of noise levels in the 125 Hz band. Levels in the three monitoring regions during (**a**) 2013 (**b**) 2014.; Levels at individual northern North Sea monitoring sites during (**c**) 2013 (**d**) 2014. Abbreviations refer to monitoring region: Celtic Sea (CS), northern North Sea (NNS), and southern North Sea (SNS). NNS data in (**a,b**) are mean probability densities computed over individual sites shown in (**c,d**).

For the majority of monitoring sites, the noise level distributions were within similar ranges when considered with respect to the 1/3-octave frequency bands at 63 and 125 Hz currently designated for MSFD monitoring. The empirical probability distributions of noise levels in the 125 Hz band are shown for 2013 (Fig. 3a) and 2014 (Fig. 3b) for each monitoring region. The modes of these distributions lie between ~84–95 dB re 1 μPa, with noise levels largely between 80 and 110 dB re 1 μPa (Fig. 3). The sites in the Celtic Sea and southern North Sea had substantial periods when noise levels were low enough for flow noise to dominate at 125 Hz. This resulted in steps in the left tail of the distribution (indicated by arrows in Fig. 3a,b) corresponding to slack and flow periods of the tidal cycle. Note that since flow noise is caused by turbulent flow around the hydrophone, it is dependent on the orientation and positioning of the recorder in the water column: slight differences in the deployment conditions likely led to the consistent difference in the left tail of the distribution at the Celtic Sea site between 2013 (Fig. 3a) and 2014 (Fig. 3b).

The greater number of monitoring stations in the northern North Sea enabled an assessment of finer scale differences within the region among a number of sites (Fig. 3c,d), although deployments were limited to periods between June and November (Table S1). Overall, the noise level distributions are broadly similar, with modes in the range ~90–95 dB re 1 μPa, and noise levels largely within the range 80–120 dB re 1 μPa. An exception is site NNS3, which is located in the relatively sheltered waters of the inner Moray Firth (Fig. 1). While this area does sustain noise from shipping^31^, levels of vessel traffic are relatively low compared to the main shipping routes along the east coast of Scotland. The shallower bathymetry will also result in comparatively poor acoustic propagation, attenuating the contribution of shipping noise from traffic in the North Sea. These results highlight how local conditions can lead to deviations from wider trends in a monitoring region.

### Summary metrics of noise level

For each of the three monitoring regions, four summary metrics of noise level were computed: the mode, median, 90^th^ percentile and RMS level (Table 1). The rank order of noise levels among monitoring regions was consistent at all frequencies assessed for the mode, median, and 90^th^ percentile (Table 1). For these metrics, the northern North Sea had the highest noise levels at 125, 250, and 500 Hz, while the southern North Sea had the highest noise levels in the 63 Hz band (due to persistent, localised tonal noise from the nearby power station; Fig. 2). The Celtic Sea had the lowest noise levels at 125 Hz and below, and the southern North Sea had the lowest noise levels at 250 Hz and above. The consistency in the rank order among these metrics suggest they are robust indicators of the distributions they represent.

The RMS level is not a robust metric, and is highly sensitive to outliers in the noise level distribution^34^, since it represents the mean computed before noise levels are converted to decibels (i.e. log transformed). Nevertheless, this is currently the metric recommended for use as an environmental indicator under the EU MSFD^28^. In this study, the RMS level exceeded the 90^th^ percentile at all frequencies and in all monitoring regions (Table 1). For the Celtic Sea and the southern North Sea, the RMS level exceeded the 97^th^ percentile. Due to noise caused by colliding fishing gear, the southern North Sea location had the highest noise levels at 63, 125, and 250 Hz. At 500 Hz, the northern North Sea had the highest RMS level noise levels, possibly due to the greater presence of vessel traffic at the recording sites.

### Power to detect statistically significant trends

**Figure 4:**
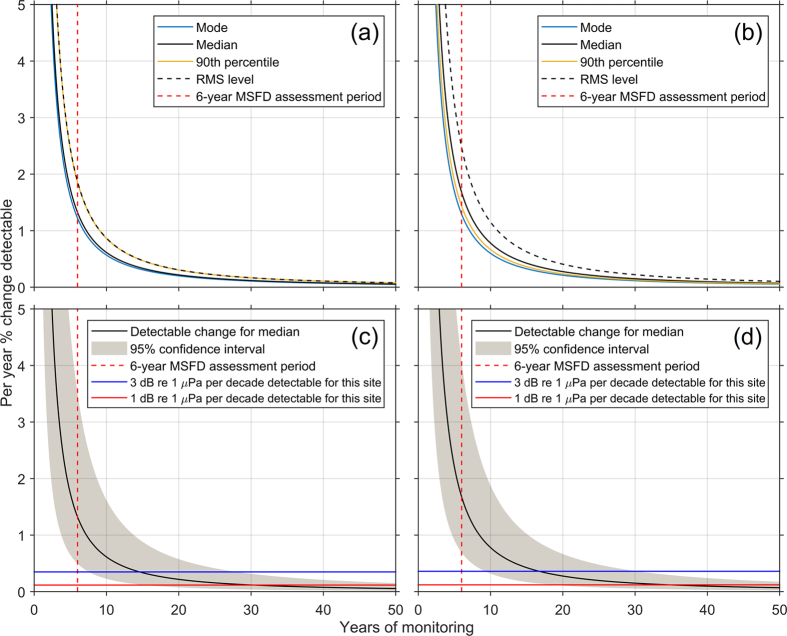
Statistical power to detect significant trends in the 125-Hz third-octave band based on monthly summary metrics for (**a**) southern North Sea and (**b**) Celtic Sea; and for the monthly median including 95% confidence intervals for (**c**) southern North Sea and (**d**) Celtic Sea.

The analysis of statistical power to detect trends revealed differences among the monthly summary metrics assessed with regard to the duration of monitoring required to detect trends, both at the southern North Sea (Fig. 4a) and the Celtic Sea (Fig. 4b) sites. The time series for the northern North Sea were too brief to carry out the power analysis on a monthly basis. Note that since the analysis did not account for several gaps between deployments in the time series, these results represent a lower bound on trend detectability. Using the mode required the shortest monitoring duration to detect a given trend, and the RMS level required the greatest (Fig. 4a,b; Table S3). However, it should be noted that the magnitude of change in noise level in general would be expected to be greater for the 90^th^ percentile and the RMS level than for the mode or the median, since these metrics are more sensitive to changes in the right tail of the probability distribution (i.e. higher noise levels), which are associated with anthropogenic noise sources such as shipping. In other words, because the magnitude of change in noise level is anticipated to be larger for the 90^th^ percentile and RMS level, these metrics may be more powerful for detection of trends in anthropogenic noise despite being less sensitive to trends when compared to the mode or median. These results demonstrate that the choice of summary metric affects the statistical power to detect trends in the time series, and that in choosing a metric, consideration should be given to the management objective.

For continuous monitoring over a period of 6 years (the period of the assessment cycle for the MSFD), the annual percentage trend that would be detectable varied from 1.2% to 2.5%, depending on location and summary metric (Table S3; Fig. 4a,b). This amounts to a 6-year trend of 7.3% to 14.9%, which corresponds to 6.4–15.2 dB re 1 μPa for these sites (Table S3).

Figure 4c,d show the detectable annual change in the median (metric chosen for the purpose of comparison with the Andrew *et al*. Northeast Pacific study^11^) with the corresponding 95% confidence interval, as well as the percentage annual change corresponding to trends of 1 and 3 dB re 1 μPa per decade. To detect a 1 dB re 1 μPa per decade trend would require 34.8 years, 95% C.I. [19.5, 62.1], at the Celtic Sea site, and 30.3 years, 95% C.I. [16.0, 57.5], at the southern North Sea site (Table S3). Further monitoring to extend the time series at these sites would decrease the magnitude of the confidence intervals.

## Discussion

Environmental management of underwater noise pollution is greatly constrained by a lack of baseline data on noise levels. This limits the ability of managers to make informed decisions at a range of scales, from the regulation of individual developments through to large-scale ecosystem-based management via legislative instruments such as the MSFD. This study presents the first coordinated national effort to assess ambient noise for management applications, providing data on baseline noise levels in UK waters. While the data are not year round at several of the sites, these data nonetheless provide considerable spatial coverage, and establish baseline levels for the assessment of future trends.

The measurements revealed considerable differences in exposure to anthropogenic noise (Fig. 2). Spectra from the Celtic Sea site indicated that the acoustic environment was almost undisturbed by anthropogenic activity (Fig. 2a), despite being positioned 15 km to the east of a convergence of shipping lanes. Compared to a recent study^35^ during 2012–2013 in Falmouth Bay (on the southern side of the Cornish peninsula), median noise levels at the Celtic Sea site were higher in the 63 Hz band (likely due to flow noise) and lower in the 125 Hz band. Other sites sustained anthropogenic noise from frequent ship passages, fishing activity (Fig. 2b,c) and onshore sources (Fig. 2b). The range of these observations raises questions around how to formulate indicators of anthropogenic noise levels and how best to develop future monitoring networks that are representative of coastal soundscapes. Noise levels in particular frequency bands (e.g. as currently defined for the MSFD^36^) do not in themselves indicate levels of noise pollution (as opposed to levels of natural sound), as evidenced by the lack of anthropogenic noise sources at the Celtic Sea location (Fig. 2a). Natural background noise (e.g. from weather processes and biotic sources) varies over time and space^37^, and trends in this natural component are not the target of noise management. Nevertheless, for a given rise in anthropogenic noise sources, noise levels in previously undisturbed sites would increase substantially more than locations that are already exposed to human activity. This greater responsiveness and sensitivity of undisturbed sites to changes in anthropogenic pressure means they could serve as useful indicators of environmental change. The monitoring of noise levels which does not explicitly discriminate between natural and anthropogenic noise (e.g. via signal processing techniques) is also supported by the fact that the differences between the noise level distributions observed (Fig. 3) were driven by identifiable sources of human activity (mainly shipping), rather than the contribution of natural noise sources. However, the current choice of MSFD frequency bands at 63 Hz and 125 Hz may inadequately reflect the risk of acoustic masking^38^, and can be contaminated by flow noise^31^, and higher frequency bands (e.g. at 250 or 500 Hz) appear to better correlate with broadband levels of shipping noise^31^.

To effectively monitor levels and track trends in underwater noise pollution, metrics are needed which are statistically robust and which are relevant to the assessment of potential impact on marine life. While a number of threshold levels for injury and disturbance have been proposed for acute noise exposure for particular taxa^16^,^39^,^40^, uncertainty over the effects of noise at the ecosystem scale limits the ability to formulate absolute thresholds for ecologically sustainable noise levels. Efforts to address this knowledge gap will need to consider the differential susceptibility to noise exposure thus far observed among different taxa^41^,^42^, the complexities of secondary and indirect effects of noise on ecosystem functioning^25^,^26^, and the differing physical properties of sound that aquatic species use to hear^43^. Until these uncertainties are addressed, progress can be made by establishing monitoring programmes to track levels of noise pollution, and by ensuring that metrics used to describe noise levels are pertinent to assessing the risk of impact to marine life. The current recommendation for the MSFD is to use the RMS level^28^, but this metric is strongly influenced by outliers in the distribution^34^, and so can be skewed away from the general trend in noise levels by a few high amplitude but unrepresentative events in the time series. This effect was clear in the levels observed in this study (Table 1), where the RMS level exceeded the 90^th^ percentile in all cases, and exceeded the 97^th^ percentile for several locations and frequencies. By contrast, percentile-based metrics and the mode are robust to such outliers. Percentile-based metrics are also directly related to the temporal distribution of noise levels, which makes them appropriate for assessing the risk of acoustic masking^21^,^44^, as well as being more straightforward to interpret and communicate to policymakers. In terrestrial acoustics, the 90^th^ percentile (also known as the L_10_ exceedance level: the level exceeded 10% of the time) is used as an indicator of intermittent anthropogenic noise^45^. We suggest that this metric (or a similar percentile, e.g. the 95^th^) is also appropriate for tracking levels of anthropogenic noise in the marine environment.

Trend detection is a critical aspect of noise monitoring programmes, both to identify long-term environmental change, and to assess the efficacy of management actions to reduce noise. Indeed, the current MSFD Indicator for ambient noise is defined according to trends in noise level^36^. Even if such environmental indicators of underwater noise pollution are ultimately defined according to absolute thresholds, trend detection could still help to evaluate progress towards such targets. Using methods developed for trend detection in environmental time series^46^, a power analysis indicated that the minimum trend in noise level that monitoring at the southern North Sea and Celtic Sea sites could detect was between 6.4 and 15.2 dB re 1 μPa (depending on location and summary metric) in the 125-Hz 1/3 octave band over a continuous 6-year monitoring period (the duration of the MSFD assessment cycle; Fig. 4). This rate of change is substantially greater than that observed in historic recordings from the Northeast Pacific, which indicate trends of ≤1 dB re 1 μPa per decade at these frequencies between the 1960 s and 1990 s, based on the median level^11^. In other words, unless trends in noise levels are several times greater in magnitude (~5–11 dB re 1 μPa per decade, depending on metric) than those observed historically for the Northeast Pacific (1 dB re 1 μPa per decade), monitoring programmes are unlikely to be able to detect upward or downward trends over the 6-year timescale of the MSFD assessment cycle. Detection of a 1 dB re 1 μPa per decade trend is projected to require 34.8 years, 95% C.I. [19.5, 62.1], at the Celtic Sea site, and 30.3 years, 95% C.I. [16.0, 57.5], at the southern North Sea site (Table S3). Given the ambition of the EU MSFD to assess and achieve ‘Good Environmental Status’ with respect to underwater noise by 2020, these results imply that the MSFD Indicator for ambient noise should be redefined in terms of absolute noise levels (e.g. thresholds), rather than the present trend-based Indicator.

The power analysis also revealed differences in the performance of the four summary metrics used to calculate the monthly averages (mode, median, 90^th^ percentile, and RMS level). The mode yielded the most powerful trend detection, and the RMS level the least, although it is important to note that the magnitude of change is likely to be greater for the 90^th^ percentile and RMS level compared to the mode, which may in practice outweigh the differences in sensitivity for trend detection. Regardless of which metric is used to characterise trends in underwater noise levels, observations in other areas of environmental science have shown that long-term, often multi-decadal monitoring is required to detect significant trends in environmental variables, and that consistency in the monitoring instrumentation, sensor placement, and continuity of the time series have a strong influence on the statistical power of monitoring programmes^46^.

A further question for noise monitoring programmes is how to target monitoring effort. While it may seem advantageous to target monitoring effort at those locations with the greatest power to detect trends, such sites are likely to have lower variance in noise levels (Eq. 1), which may be associated with lower anthropogenic influence (e.g. from passing vessels) and lead to bias toward less disturbed areas. The site selection process should instead consider which locations are most informative for management purposes. Given that shipping is the most widespread source contributing to increased noise levels, monitoring of major shipping lanes could serve as a bellwether for wider trends in shipping noise pollution. Another approach is to consider the distribution of species that may be affected by noise, and to target hotspots of overlap between high animal density and anthropogenic noise levels^47^, or to prioritise areas of high animal density and low anthropogenic pressure^48^. To build a monitoring programme that can effectively inform management action, we suggest that a combination of these approaches will be required. Targeting monitoring in key habitats for protected or ecologically important species (e.g. Marine Protected Areas; MPAs) ensures that the outcomes of mitigation measures to protect priority areas can be adequately assessed. This can also lead to efficiencies in monitoring effort as many MPAs are already the target of monitoring programmes for animal abundance or other stressors. Trends at these priority sites need to be set in the context of wider trends in noise pollution. In the case of shipping, such trends could be efficiently monitored at sites where major shipping routes are focussed, e.g. in Western Europe, the Strait of Dover and the Strait of Gibraltar.

It has been suggested that mapping of noise levels using acoustic models could provide a more comprehensive indicator of marine noise pollution, and reduce the number of monitoring stations needed^28^. However, although noise maps based on human activity data (e.g. AIS ship-tracking data) have been developed^49^,^50^, such models have yet to be thoroughly validated using field measurements^17^. The present study has highlighted the issue of onshore noise sources contributing to marine soundscapes, which cannot be modelled using current techniques. There are also other major uncertainties in these geospatial models that have yet to be resolved, for example: uncertainties in noise levels and frequency characteristics of noise sources; lack of geospatial data on many noise sources (e.g. small vessels^51^); and uncertainty in acoustic propagation models used to predict how noise spreads in the environment (which depend on the quality of available geophysical and oceanographic data^52^). Unless such uncertainties are adequately addressed, field measurements will present the primary means by which noise levels can be quantitatively assessed. Nevertheless, geospatial models can play an important role in identifying priority monitoring locations^47^,^48^ to help target monitoring effort.

## Methods

Data were collected using autonomous underwater acoustic recorders during 2013 and 2014 in three coastal monitoring areas: the Celtic Sea, the northern North Sea, and the southern North Sea (see Fig. 1). Data were collected under three separate programmes and collated centrally for analysis. Southern North Sea data were recorded as part of the EDF Energy New Nuclear Build marine studies programme from February to September 2013, using a Jasco Autonomous Multichannel Acoustic Recorder (AMAR) G3. Northern North Sea deployments formed part of the Marine Scotland East Coast Marine Mammal Acoustic Study (ECOMMAS), and were made in the summers of 2013 and 2014 (August to October and June to September respectively) using Wildlife Acoustics SM2M, while the Celtic Sea deployments were made at the Wave Hub test site^53^ and recorded almost continuously during 2013 and 2014, also using the Wildlife Acoustics SM2M. Data from site NNS6 (Fig. 1) were contaminated with noise from a nearby acoustic deterrent device (ADD), and were omitted from further analysis. Full details of deployment times, location coordinates, deployment depths, and recorder settings are provided in the Supplementary Material.

**Figure 5:**
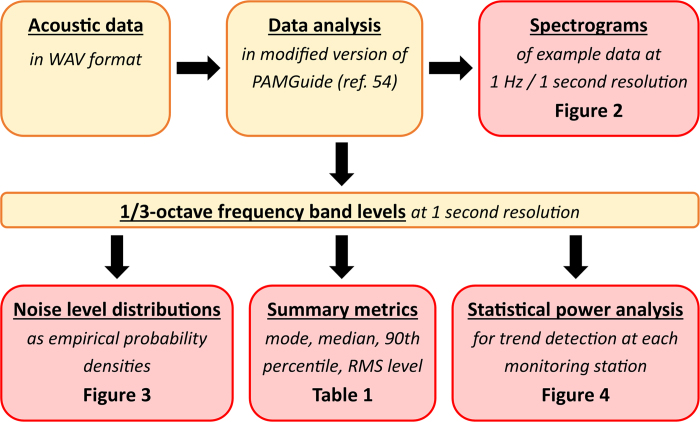
Flow diagram illustrating the data processing stages undertaken in the analysis.

In total, 13.4 TB of acoustic data were collected by the field programmes, constituting over 6 years of monitoring. The data analysis was carried out in several stages as shown in Fig. 5, using a modified version of PAMGuide^54^, which corrected for the frequency sensitivity of the acoustic recorders. Example data were analysed as power spectral densities at 1-second resolution, yielding spectrograms at 1-Hz frequency resolution for the purposes of identifying noise sources. While spectrograms have fine-scale frequency resolution suitable for source identification, 1/3-octave frequency bands are more appropriate to describe distributions and trends in noise levels as they combine noise levels over a standardised frequency range into a single metric^54^. For this purpose, the entire dataset was analysed at 1-second resolution in 1/3-octave frequency bands from 50 Hz to 1000 Hz. Four frequency bands were selected for detailed analysis: those corresponding to the MSFD monitoring frequencies (63 Hz and 125 Hz), and two higher frequency bands at 250 Hz and 500 Hz, which have been shown to correlate well with levels of broadband shipping noise^31^. Empirical probability distributions of noise levels in these frequency bands were computed using a kernel smoothing density function as described by Merchant *et al*.^34^. Finally, several statistical metrics of these distributions were computed for each region to provide indicative baseline levels: the median, the RMS level, the mode and the 90^th^ percentile. Metrics for the northern North Sea were aggregated by first computing the metrics for each of the monitoring sites, and then taking the median value in each case.

An analysis was also conducted to assess the statistical power of the monitoring locations to detect significant trends in noise levels. Following the methodology described by Weatherhead *et al*.^46^, the magnitude of change detectable per year, |ω|, for a given number of years of monitoring, *n*, is given by:


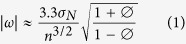


where *σ*_*N*_ is the standard deviation of the noise expressed as a percentage (the noise being the portion of the data unexplained by the trend), and ∅ is the autocorrelation of the time series, where it is assumed that |∅| < 1. So for example, a result of |ω| = 0.2 would indicate that a 0.2% annual upward or downward trend could be detected at the 95% confidence level and with a probability of 0.90, over the specified number of years, *n*.

Similarly, the number of years, n^*^, required to detect a trend of a given magnitude, |ω_0_|, is given by


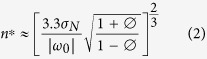


The 95% confidence interval for n^*^ is computed using the uncertainty factor, *B*, defined as


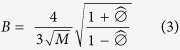


where *M* is the number of months of data collected, and 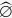 is the estimated autocorrelation, ∅, over the projected monitoring period, based on the available data. The 95% confidence interval is then given by 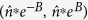, where 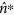 is computed using Eq. 2.

## Additional Information

**How to cite this article**: Merchant, N. D. *et al*. Underwater noise levels in UK waters. *Sci. Rep*. **6**, 36942; doi: 10.1038/srep36942 (2016).

**Publisher’s note:** Springer Nature remains neutral with regard to jurisdictional claims in published maps and institutional affiliations.

## Supplementary Material

Supplementary Information

## Figures and Tables

**Table 1:** Summary metrics of noise level for UK monitoring regions, with data for the North Sea sites represented by the median value among the nine monitoring sites.

	Monitoring region	63 Hz	125 Hz	250 Hz	500 Hz
Mode	Celtic Sea	75.8	83.2	88.4	91.5
	North Sea	90.0	92.0	94.5	94.3
	Southern North Sea	94.0	87.0	72.7	82.3
Median	Celtic Sea	82.0	83.3	87.1	89.7
	North Sea	90.5	93.6	95.5	94.6
	Southern North Sea	94.7	86.0	78.9	83.5
90^th^ percentile	Celtic Sea	93.2	93.3	96.0	96.9
	North Sea	100.3	103.5	103.9	103.3
	Southern North Sea	102.0	96.5	94.3	93.3
RMS level	Celtic Sea	101.6	102.3	102.9	99.9
	North Sea	101.8	103.8	104.5	104.2
	Southern North Sea	110.8	113.1	113.3	104.9

Metrics represent all data from each site (i.e. over both monitoring years where applicable). All metrics are sound pressure levels in the corresponding 1/3-octave frequency band, in units of dB re 1 μPa.
