# Supplementary material for: Underwater noise levels in UK waters
Source: Sci Rep. 2016 Nov 10;6:36942. doi: 10.1038/srep36942 (PMC5103265; doi:10.1038/srep36942)
Supplement: Supplementary Information [file srep36942-s1.docx]

**Underwater noise levels in UK waters**

*Supplementary information: deployment details and additional data*

Nathan D. Merchant^1^*, Kate L. Brookes^2^, Rebecca C. Faulkner^1^, Anthony W. J. Bicknell^3,4^,

Brendan J. Godley^3,4^, Matthew J. Witt^3^

^1^Centre for Environment, Fisheries & Aquaculture Science (Cefas), Lowestoft, UK

^2^Marine Scotland Science, Aberdeen, UK

^3^Environment and Sustainability Institute, University of Exeter, Penryn, UK

^4^Centre for Ecology and Conservation, University of Exeter, Penryn, UK

*Corresponding author (email: nathan.merchant@cefas.co.uk).

Table S1. Deployment locations and periods.

| **Area** | **Location ID** | **Lat** | **Lon** | **Depth (m)** | **Duty cycle** | **Sample rate** | **Data start date** | **Data end date** |
| --- | --- | --- | --- | --- | --- | --- | --- | --- |
| **Celtic Sea** | CS | 50.3392 | -5.625366667 | 50 | 30 min on, 30 min off | 96 kHz | 01/01/2013 | 03/01/2013 |
|  |  | 50.3392 | -5.625366667 | 50 | 30 min on, 30 min off | 96 kHz | 03/01/2013 | 12/03/2013 |
|  |  | 50.3392 | -5.625366667 | 50 | 30 min on, 30 min off | 96 kHz | 20/03/2013 | 12/06/2013 |
|  |  | 50.3392 | -5.625366667 | 50 | 30 min on, 30 min off | 96 kHz | 26/06/2013 | 28/08/2013 |
|  |  | 50.3392 | -5.625366667 | 50 | 30 min on, 30 min off | 96 kHz | 28/08/2013 | 14/11/2013 |
|  |  | 50.3392 | -5.625366667 | 50 | 30 min on, 30 min off | 96 kHz | 16/11/2013 | 31/01/2014 |
|  |  | 50.3392 | -5.625366667 | 50 | 30 min on, 30 min off | 96 kHz | 29/04/2014 | 31/07/2014 |
|  |  | 50.3392 | -5.625366667 | 50 | 30 min on, 30 min off | 96 kHz | 05/08/2014 | 13/10/2014 |
|  |  | 50.3392 | -5.625366667 | 50 | 30 min on, 30 min off | 96 kHz | 24/11/2014 | 31/12/2014 |
| **Southern North Sea** | SNS | 52.221833 | 1.63275 | 7 | 2 min on, 8 min off | 48 kHz | 09/02/2013 | 05/06/2013 |
|  |  | 52.221833 | 1.63275 | 7 | 2 min on, 8 min off | 48 kHz | 05/06/2013 | 27/09/2013 |
| **North Sea** | NS1 | 58.26933342 | -3.318194357 | 23 | 10 min on, 10 min off | 96 kHz | 10/08/2013 | 14/10/2013 |
|  | NS2 | 57.97570306 | -3.53583982 | 49 | 10 min on, 10 min off | 96 kHz | 10/08/2013 | 25/09/2013 |
|  | NS3 | 57.70668324 | -3.81071818 | 16 | 10 min on, 10 min off | 96 kHz | 10/08/2013 | 15/10/2013 |
|  | NS4 | 57.74148223 | -3.03881625 | 22 | 10 min on, 10 min off | 96 kHz | 10/08/2013 | 12/10/2013 |
|  | NS5 | 57.71134639 | -2.130122221 | 37 | 10 min on, 10 min off | 96 kHz | 10/08/2013 | 07/10/2013 |
|  | *NS6** | *57.38019025* | *-1.828363841* | 22 | *10 min on, 10 min off* | *96 kHz* | *10/08/2013* | *12/10/2013* |
|  | NS7 | 56.9469442 | -2.176712707 | 28 | 10 min on, 10 min off | 96 kHz | 10/08/2013 | 03/10/2013 |
|  | NS8 | 56.49979842 | -2.379894724 | 49 | 10 min on, 10 min off | 96 kHz | 10/08/2013 | 11/10/2013 |
|  | NS9 | 56.2578941 | -2.499311904 | 43 | 10 min on, 10 min off | 96 kHz | 10/08/2013 | 18/10/2013 |
|  | NS10 | 55.92919788 | -2.177105101 | 33 | 10 min on, 10 min off | 96 kHz | 10/08/2013 | 28/11/2013 |
|  | NS1 | 58.26933342 | -3.318194357 | 23 | 10 min on, 20 min off | 96 kHz | 25/06/2014 | 05/10/2014 |
|  | NS2 | 57.97570306 | -3.53583982 | 49 | 10 min on, 20 min off | 96 kHz | 25/06/2014 | 25/09/2014 |
|  | NS3 | 57.70668324 | -3.81071818 | 16 | 10 min on, 20 min off | 96 kHz | 25/06/2014 | 29/09/2014 |
|  | NS4 | 57.74148223 | -3.03881625 | 22 | 10 min on, 20 min off | 96 kHz | 25/06/2014 | 12/09/2014 |
|  | NS5 | 57.71134639 | -2.130122221 | 37 | 10 min on, 20 min off | 96 kHz | 25/06/2014 | 14/09/2014 |
|  | *NS6** | *57.38019025* | *-1.828363841* | 22 | *10 min on, 20 min off* | *96 kHz* | *25/06/2014* | *05/10/2014* |
|  | NS7 | 56.9469442 | -2.176712707 | 28 | 10 min on, 20 min off | 96 kHz | 25/06/2014 | 24/09/2014 |
|  | NS8 | 56.49979842 | -2.379894724 | 49 | 10 min on, 20 min off | 96 kHz | 25/06/2014 | 12/09/2014 |
|  | *NS9** | *56.2578941* | *-2.499311904* | 43 | *10 min on, 20 min off* | *96 kHz* | *25/06/2014* | *12/09/2014* |
|  | NS10 | 55.92919788 | -2.177105101 | 33 | 10 min on, 20 min off | 96 kHz | 25/06/2014 | 12/09/2014 |

* Data unusable.

Table S2. Summary metrics for all sites.

| **Site** | **Mode** | | | | **Median** | | | | **90th percentile** | | | | **RMS level** | | | |
| --- | --- | --- | --- | --- | --- | --- | --- | --- | --- | --- | --- | --- | --- | --- | --- | --- |
| *Frequency:* | *63* | *125* | *250* | *500* | *63* | *125* | *250* | *500* | *63* | *125* | *250* | *500* | *63* | *125* | *250* | *500* |
| **CS** | **75.8** | **83.2** | **88.4** | **91.5** | **82.0** | **83.3** | **87.1** | **89.7** | **93.2** | **93.3** | **96.0** | **96.9** | **101.6** | **102.3** | **102.9** | **99.9** |
| **SNS** | **94.0** | **87.0** | **72.7** | **82.3** | **94.7** | **86.0** | **78.9** | **83.5** | **102.0** | **96.5** | **94.3** | **93.3** | **110.8** | **113.1** | **113.3** | **104.9** |
| NNS1 | 90.5 | 92.3 | 93.0 | 90.9 | 90.5 | 93.0 | 94.1 | 93.2 | 97.9 | 100.5 | 102.1 | 101.1 | 94.6 | 97.6 | 100.5 | 102.4 |
| NNS2 | 90.1 | 90.6 | 90.9 | 90.3 | 91.2 | 92.2 | 91.9 | 90.6 | 100.1 | 100.9 | 99.2 | 97.6 | 98.7 | 99.6 | 99.1 | 97.2 |
| NNS3 | 82.9 | 86.3 | 89.5 | 91.4 | 85.3 | 89.0 | 92.0 | 92.7 | 100.5 | 104.1 | 108.4 | 108.5 | 103.9 | 103.8 | 106.5 | 106.2 |
| NNS4 | 90.0 | 91.7 | 93.0 | 94.7 | 92.3 | 93.7 | 94.4 | 94.6 | 104.2 | 104.4 | 103.9 | 103.3 | 107.2 | 106.3 | 104.5 | 103.8 |
| NNS5 | 84.1 | 92.0 | 94.5 | 93.3 | 86.5 | 93.6 | 96.0 | 94.6 | 98.1 | 103.5 | 106.0 | 104.4 | 98.2 | 105.0 | 106.2 | 108.8 |
| NNS7 | 79.6 | 89.9 | 96.1 | 95.8 | 81.8 | 90.9 | 96.4 | 96.8 | 93.9 | 98.3 | 103.5 | 104.2 | 91.8 | 96.9 | 101.7 | 114.8 |
| NNS8 | 91.3 | 93.3 | 94.6 | 94.8 | 93.3 | 95.1 | 95.7 | 95.2 | 103.8 | 105.3 | 106.1 | 104.7 | 103.8 | 107.2 | 107.9 | 106.2 |
| NNS9 | 89.0 | 93.2 | 95.0 | 94.9 | 90.4 | 94.5 | 95.5 | 95.4 | 100.3 | 102.9 | 103.6 | 102.9 | 102.8 | 105.5 | 105.5 | 104.2 |
| NNS10 | 90.6 | 95.3 | 95.8 | 94.3 | 92.4 | 95.9 | 95.9 | 94.2 | 104.9 | 105.4 | 104.5 | 103.0 | 101.8 | 102.6 | 102.0 | 100.7 |
| **NNS MEDIAN** | **90.0** | **92.0** | **94.5** | **94.3** | **90.5** | **93.6** | **95.5** | **94.6** | **100.3** | **103.5** | **103.9** | **103.3** | **101.8** | **103.8** | **104.5** | **104.2** |

Table S3 Power analysis coefficients for metrics computed on a monthly basis from the raw 1-second time resolution data in the 125-Hz frequency band, based on Equation 1 (see main text). Levels given in dB re 1 µPa are based on the % change from the value of each metric as presented in Table S2.

|  | **Metric** | $\boldsymbol{\emptyset}$ | $\boldsymbol{\sigma}_{\boldsymbol{N}}$  **[%]** | **B** | $\left\vert\boldsymbol{\omega} \right\vert$ **for 6 years of continuous monitoring**  **[%]** | **Total detectable trend over 6 years [%]** | $\left\vert\boldsymbol{\omega} \right\vert$ **for 6 years of continuous monitoring**  **[dB re 1 µPa]** | **Total detectable trend over 6 years [dB re 1 µPa]** | ***n* years monitoring to detect 3 dB re 1 µPa per decade trend** | **95% C.I. for 3 dB trend** | ***n* years monitoring to detect 1 dB re 1 µPa per decade trend** | **95% C.I. for 1 dB trend** |
| --- | --- | --- | --- | --- | --- | --- | --- | --- | --- | --- | --- | --- |
| **CS** | mode | 0.32 | 4.12 | 0.43 | 1.3 | 7.7 | 1.1 | 6.4 | 14.0 | [9.2, 21.5] | 29.2 | [19.0, 44.7] |
|  | median | 0.56 | 3.95 | 0.58 | 1.7 | 10.1 | 1.4 | 8.4 | 16.7 | [9.4, 29.8] | 34.8 | [19.5, 62.1] |
|  | 90th | 0.63 | 3.02 | 0.65 | 1.4 | 8.6 | 1.3 | 8.0 | 16.3 | [8.5, 31.0] | 33.8 | [17.7, 64.5] |
|  | RMS | 0.67 | 4.90 | 0.69 | 2.5 | 14.9 | 2.5 | 15.2 | 24.9 | [12.5, 49.5] | 51.8 | [26.0, 103.0] |
| **SNS** | mode | 0.30 | 3.99 | 0.61 | 1.2 | 7.3 | 1.1 | 6.4 | 13.9 | [7.6, 25.5] | 29.0 | [15.8, 53.1] |
|  | median | 0.35 | 4.10 | 0.64 | 1.3 | 7.9 | 1.1 | 6.8 | 14.6 | [7.7, 27.6] | 30.3 | [16.0, 57.5] |
|  | 90th | 0.31 | 5.97 | 0.62 | 1.9 | 11.1 | 1.8 | 10.7 | 19.7 | [10.7, 36.6] | 41.1 | [22.2, 76.0] |
|  | RMS | 0.04 | 8.02 | 0.46 | 1.9 | 11.2 | 2.1 | 12.7 | 22.0 | [13.9, 34.9] | 45.8 | [28.9, 72.7] |
